# Powassan virus persistence after acute infection

**DOI:** 10.1128/mbio.00712-23

**Published:** 2023-06-20

**Authors:** Sam R. Telford, Anne L. Piantadosi

**Affiliations:** 1 Department of Infectious Disease and Global Health; Tufts Lyme Disease Initiative, Tufts University, North Grafton, Massachusetts, USA; 2 Department of Pathology and Laboratory Medicine, Emory University School of Medicine, Atlanta, Georgia, USA; University of Colorado School of Medicine, Aurora, Colorado, USA

**Keywords:** Powassan encephalitis, post-acute neurological sequelae, mouse model, arboviral encephalitis

## Abstract

Survivors of Powassan encephalitis often have persistent neurological disease. A new mouse model replicates some elements of the human disease and demonstrates the presence of viral RNA in the brain as well as myelitis more than 2 mo after the acute infection. The related tick-borne encephalitis and West Nile Neuroinvasive Disease (WNND) also have common neurological sequelae, and models for these better-studied diseases provide evidence for prolonged virus, RNA, and inflammation in some cases, in addition to damage from the acute encephalitic disease. A better understanding of the biological basis for persistent signs and symptoms after Powassan encephalitis, currently a rare disease, could benefit from further studies of the more prevalent flaviviral encephalitides.

## Text

Powassan encephalitis is recognized as a severe arboviral infection in humans, with both high mortality in acute infection and residual symptoms in many individuals who recover. In this issue, Scroggs and colleagues (1) report viral persistence after experimental infection of 6-wk-old C57/BL6 mice with a low dose (3 log focus forming units, intraperitoneal) of Powassan virus (POWV). Acute infection was associated with severe disease and death in 17% of mice, comparable to the estimated human case fatality rate (CFR) of Powassan encephalitis (12%). Brain tissue from the animals who died during acute infection was characterized by widespread inflammation, detection of viral RNA, and isolation of the infectious virus, hallmarks of a severe acute infection. Among the mice that survived acute infection, few had persistent symptoms or clinical signs of disease. However, there was notable inflammation and detectable viral RNA in brain tissue from mice sacrificed at 21 d after inoculation; the infectious virus was not present. Even some of the mice that were sacrificed at 56 d and 84 d had persistent viral RNA (60% and 40% of mice, respectively) and inflammation (20% and 0% of mice, respectively) detected in brain tissue. Interestingly, there was also evidence of myelitis (spinal cord inflammation) in 20% of mice sacrificed at 84 d. Based on this rigorous investigation, the authors conclude that persistent RNA and chronic inflammation, rather than active viral infection, may account for long-term neurological symptoms in patients with Powassan encephalitis. Their model provides an important foundation for better understanding the chronic debilitation that characterizes survivors of Powassan encephalitis and investigating potential interventions. Here, we comment on the generality of persistent disease after arboviral encephalitis, with a specific emphasis on the tick-borne encephalitis complex viruses and on West Nile virus, a mosquito-borne infection that is common in regions where people are at risk of acquiring Powassan encephalitis.

POWV infection in humans has been said to be “notable for the severity of both the acute disease and the long-term sequelae” (2). Acute Powassan disease symptoms include fever and fatigue, and although mild disease has been described, 95% of recognized cases are neuroinvasive (3). Most patients with neuroinvasive disease have encephalitis or meningoencephalitis, characterized by headache, confusion, weakness, focal deficits, and seizures (4). Brain imaging most commonly demonstrates involvement of the basal ganglia and cerebellum, as well as brainstem and cortex. Spinal cord involvement is rarely described. Neuropathology studies from patients with Powassan encephalitis demonstrate lymphocytic inflammation, microgliosis with microglial nodules, neuronal loss, and necrosis, all consistent with findings described by Scroggs *et al*. in the C57/BL6 mice that succumbed to acute infection; unlike the mouse model, however, infection in humans is not associated with neutrophilic inflammation. Direct neuronal infection by POWV has been observed by ISH in human brain tissue (4), similar to the Scroggs et al. study. Among 270 human cases of Powassan disease described in the literature to date, the overall mortality rate is 12%, and all deaths have occurred in individuals aged 49 and more, except for the first case described in 1959. Two studies reported an average duration of hospitalization of 18.6 d (range 4–46) and 33 d (range 9–90), and in one study most patients had residual neurological deficits at discharge, requiring inpatient rehabilitation (4).

Among survivors, post-acute sequelae of POWV infection include cognitive deficits, speech difficulty, weakness, imbalance, ophthalmoplegia, and persistent headaches. These sequelae have been reported across multiple studies, and they are estimated to occur in about one-third of individuals who survive acute POWV infection (5). However, further work is needed to define the incidence, duration, and severity of chronic sequelae, as well as factors that contribute to them. POWV RNA is readily detected in human brain tissue during acute infection (4), but it is unclear how long it persists. One patient who died more than a month after symptom onset had extensive inflammation and necrosis by brain autopsy, with possible rare viral inclusions, but no viral particles by electron microscopy (6). Similar to the Scroggs et al. study, studies of human infection have rarely detected viruses outside of the central nervous system. Further work is needed to understand potential contributions to chronic sequelae, including persistent virus, chronic inflammation, and residual neurological damage.

Can Eurasia’s greater experience with tick-borne encephalitis virus (TBEV) provide insight into post-acute sequelae of POWV? TBEV complex viruses (Flaviviridae) are an ecologically and phenotypically diverse group maintained by ticks across Eurasia; POWV is the sole member of this complex in North America. Like many arboviruses, TBE-group viruses cause clinical illness only in a minority of the infected individuals, but there may be severe morbidity and mortality among those who do develop the disease, and sequelae are common (7). TBE is the most burdensome tick-transmitted infection in Eurasia and comprises two distinct patterns of acute disease. The first is a milder biphasic febrile illness that may progress to neuroinvasive disease, with a <2% CFR. The second is severe meningoencephalitis, with CFR as great as 20–40% (8). These patterns are typically associated with distinct TBE subtypes, suggesting that they may be due to different virulence phenotypes (9). Central European encephalitis virus (CEEV) causes the first, milder disease, and Far Eastern TBE, known classically as Russian Spring Summer Encephalitis, causes the second. There is a third subtype, Siberian TBE, which causes a mainly febrile disease with 6% CFR. Despite these general patterns, both mild and severe manifestations have been ascribed to all three viral subtypes, indicating that viral factors are complemented by additional modifiers such as age, comorbidity, or infectious dose (10). Acute TBE meningoencephalitis is often associated with a transient ataxia of the upper and lower limbs, and shoulder girdle paralysis is a common sequela. About 10–30% of TBE patients with CNS disease suffer from long-lasting or permanent neuropsychological sequelae, such as headache, lack of concentration, depression, memory impairment, hearing impairment, and tinnitus (3). Myelitis also occurs and may lead to permanent paresis. Nonhuman primate models of TBE demonstrate prolonged (5 mo) encephalitis, and the isolated virus was attenuated for virulence in mouse models (11). However, very little is known about TBE persistence in human infection. TBE RNA is readily detected in brain tissue from patients with acute encephalitis, and it was found in blood and urine from one immunocompromised patient 2 mo after symptom onset (12). TBEV can also be isolated from the brain of patients with a persistent (>2 yr) form of the disease (13).

The co-endemic, mosquito-transmitted WNV, like all flavivirus infections, is subclinical in >80% of those exposed; most symptomatic patients have a self-resolving febrile illness. However, 1% of symptomatic patients develop WNND, which may present as encephalitis, meningitis, or a poliomyelitis-like syndrome. Characteristic symptoms include tremors, cerebellar ataxia, and Parkinsonism, while the polio-like form manifests as flaccid paralysis, potentially leading to quadriplegia, respiratory difficulty, and neurogenic atrophy of the skeletal muscle (14). The histological findings are nonspecific and may include perivascular cuffing and neuronal loss. Interestingly, 50% of WNND patients and 25% of WNV non-neuroinvasive febrile disease patients demonstrate persistent disease, with neuropsychological (depression, memory/cognitive, and dysfunction) and motor skill deficits (8). WNV was detected in CSF and serum >60 donset of illness in an immunocompromised patient; RT-PCR detected viral RNA, and lesions (neuronal loss, parenchymal lymphocytic infiltration, and gliosis) identified in postmortem samples of the brain (15). WNV also establishes persistent infection in NHP models (16), and WNV or its RNA persists in the brain of infected 5-wk-old C57/BL6 for as long as 180 d (17).

Overall, it is clear that many arboviral encephalitides have post-acute neurological sequelae in human infection, and animal models support persistent virus, RNA, and inflammation in TBE and WNV. The mechanisms that are the basis for post-acute sequelae of Powassan encephalitis remain poorly understood, so the mouse model described by Scroggs et al. (1) is a critical contribution. Their C57BL/6 model is characterized by persistent viral RNA and neuroinflammation in mice who survive acute infection, but the mice did not show substantial signs of disease and the authors could not isolate the virus in cell culture from day 21 onward. In another mouse model of POW, balb/c mice (5 wk old) infected by the same strain and dose of POWV (DTV-Spo), but delivered intradermally, did have replicating virus in multiple brain regions at day 24, as determined by negative-sense RNA detected by ISH (18). Thus, both C57BL/6 and balb/c mouse models could be useful for probing additional questions on the mechanisms of viral persistence as well as for testing therapeutic modalities. These models offer key improvements over cell cultures, which may support persistent low-level viral replication and expression of viral antigens, but are poorly productive of infectious virus and complicated by the production of defective virus (19). Studying the interplay between host immunogenetic factors, viral strain variability, and other potential contributors to post-acute sequelae requires robust models of disease such as the one described here.

Further study of humans is needed to determine whether post-acute sequelae of Powassan encephalitis differ from those described in WNV, TBE, and other infections. This is challenging given its low incidence compared to the thousands of annual cases of WNV and TBE. With rare diseases, every case needs to be studied carefully: Powassan encephalitis survivors should be offered enrollment in comprehensive research studies that not only define the clinical spectrum, incidence, and risk factors for post-acute sequelae (as has been done for WNV [20]) but also to probe molecular contributors such as immune dysregulation. The field would benefit from a comparative approach including other flaviviral encephalitides, and using the sum of evidence to try to better understand and ultimately treat chronic debilitation in Powassan encephalitis patients.
